# Content Analysis of YouTube Videos on Radiographic Anatomy on Dental Panoramic Images

**DOI:** 10.3390/healthcare10081382

**Published:** 2022-07-25

**Authors:** Andy Wai Kan Yeung

**Affiliations:** Oral and Maxillofacial Radiology, Applied Oral Sciences and Community Dental Care, Faculty of Dentistry, The University of Hong Kong, Hong Kong, China; ndyeung@hku.hk

**Keywords:** YouTube video, dental education, oral and maxillofacial radiology, panoramic, student-centered learning

## Abstract

The radiographic anatomy on dental panoramic images is essential knowledge for proper diagnosis and treatment planning purposes. No prior study has examined the content of YouTube videos with regard to radiographic anatomy on panoramic radiography. The objective of this study was to provide a content analysis on these videos. The initial search string was: (panoramic anatomy). An additional search was performed with the search string: (OPG landmarks). By screening the resultant videos and their related videos (recommended by YouTube as a list on the right of the screen), a total of 62 videos were screened. Videos were excluded if they were irrelevant (e.g., focusing on radiographic errors without covering the anatomy), elaborating mainly with drawings without showing the landmarks on panoramic images, duplicate videos, and non-English speaking. Finally, 38 videos were included and analyzed. Most of them showed clear panoramic images and had clear tracing or delineation of the anatomical landmarks. On average, each video described 26 landmarks, including 12.3 from the midfacial region, 8.2 from the mandible, and 5.2 from soft tissue/air space/others. The videos were of good quality in general, with some frequent shortcomings being lack of visual aid with skull and schematic diagrams, and lack of discussion on clinical relevance. The maxillary sinus was the structure mostly involved in wrong information, particularly the wrong delineation of its posterior wall.

## 1. Introduction

Panoramic radiography can be considered as one of the essential diagnostic tools for dentists. Nowadays, most panoramic machines in dental clinics generate digital radiographic images to be evaluated on computer screens [1]. The overall reject rate of panoramic radiographs across the literature was about 4% [2]. With a variety of image enhancement options available in the software, the digital armamentarium could partly overcome some of the most common reasons for the rejects, such as lack of sharpness or low contrast [3]. Besides evaluating the dentition, panoramic images could also visualize the jaw bones and midfacial structures, and hence the potential use for detecting non-dental conditions such as bone resorption under chin implants [4], osteoporosis [5,6] and Eagle syndrome [7]. YouTube videos have been utilized by students to learn endodontic and oral surgical treatments [8,9,10]. For anatomical education, a recent survey in Ireland found that around 78% of undergraduates consulted YouTube as their primary source of anatomy videos. Initiatives such as the Human Anatomy Education Channel were launched to support independent learning on YouTube with reliable videos uploaded [11]. However, most YouTube videos focused on the body trunk and the extremities but there was generally a lack of videos on head and neck anatomy [12]. Meanwhile, there are also YouTube channels on medical radiology education [13,14]. There seemed to be few organized playlists on dental radiographic anatomy, and hence an appreciation of the video contents was warranted. The aim of this study was to evaluate YouTube videos that covered the radiographic anatomy in panoramic images, to reveal the common structures described by them and any associated inaccuracies, and to identify exemplar videos as potential learning resources for students and clinicians.

## 2. Materials and Methods

**Figure 1:**
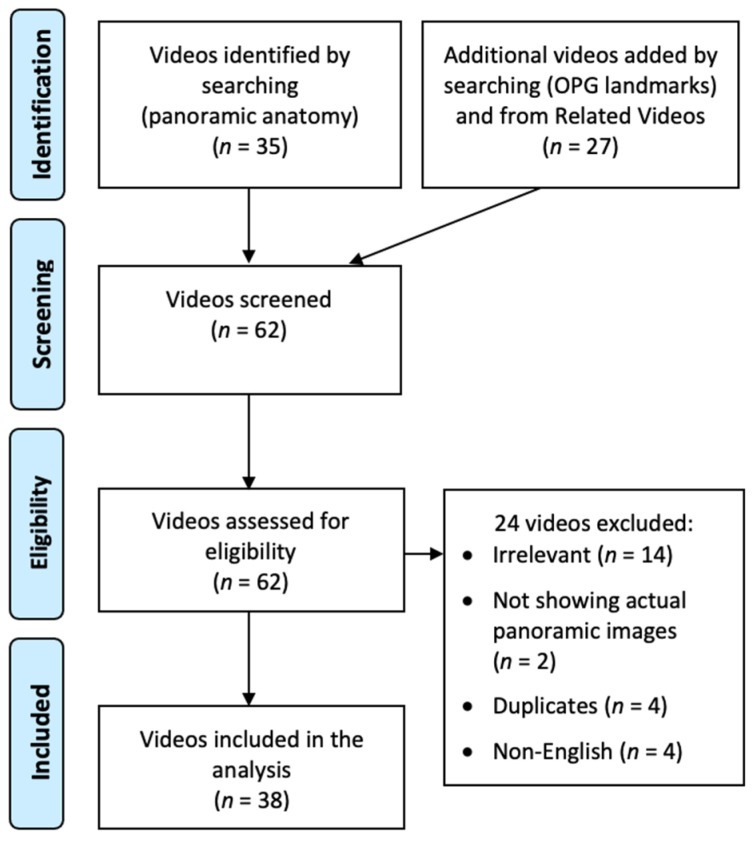
A flow chart showing the screening process of YouTube videos on panoramic anatomy.

On 1 June 2022, a video search was performed on YouTube. The initial search string was: (panoramic anatomy). The search yielded 35 videos. An additional search was performed with the search string: (OPG landmarks). By screening the resultant videos from this additional search and their related videos (recommended by YouTube as a list on the right of the screen), another 27 videos with a unique identity were identified. Hence, a total of 62 videos were screened. Exclusion criteria included irrelevance (e.g., focusing on radiographic errors without covering the anatomy), explanations mainly featuring drawings without showing the landmarks on panoramic images, duplicate videos, and non-English speaking. Figure 1 illustrates the whole video screening process. Finally, 38 videos were included and analyzed. For each included video, the following parameters were recorded: (1) Viewing metrics: view count, like count, comment count, channel subscriber count, duration (s), upload date; (2) Inaccuracies in the elaboration on the anatomical landmarks; (3) General appraisal (yes/no): clear panoramic images shown, clear tracing/delineation of landmarks, visual aid with skull, visual aid with schematic diagrams, clinical relevance of any landmarks (and their details); (4) Mid-facial landmarks; (5) Mandibular landmarks; and (6) Landmarks of soft tissues, air spaces, and others.

The number of views, likes, channel subscriber, and comments were recorded, as well as the video duration, age of video, and number of anatomical landmarks covered. Pearson correlation tests were conducted to evaluate if any of these performance metrics were significantly correlated. Two-sample t tests were performed to evaluate if the mean view count and like count were significantly different between videos with and without wrong information. A test was considered significant if *p* < 0.05.

Ethical approval was not applicable to this study.

## 3. Results

**Figure 2:**
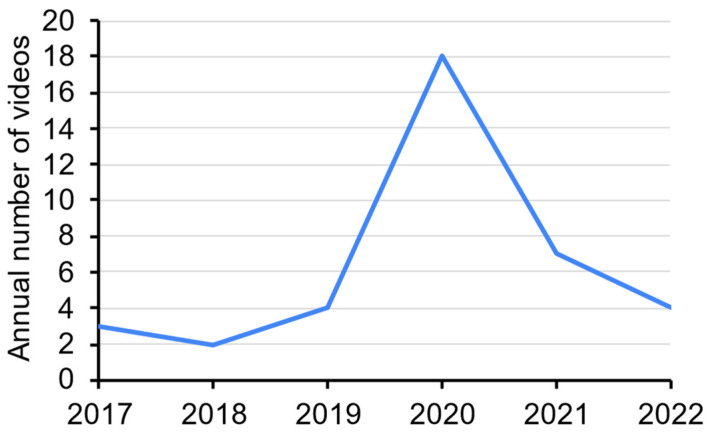
Number of YouTube videos uploaded each year on panoramic anatomy.

The details (including the web links) of the 38 videos on demonstration of panoramic radiography are listed in Supplementary Table S1. The oldest one was uploaded on 27 June 2017, whereas the most recent one was uploaded on 30 April 2022. The highest number of videos uploaded (*n* = 18) was in 2020. There was no obvious trend in the number of videos uploaded across the years (Figure 2).

The 38 videos were each viewed 7536 times on average, with 155 likes, 20 comments, and lasted for 892 s (Table 1). Comments were turned off for five videos.

For general appraisal, the majority of the videos had shown clear panoramic images (*n* = 34, 89.5%) and clear tracing/delineation of the anatomical landmarks (*n* = 29, 76.3%). On the other hand, only around one-third of them used a visual aid with a skull (*n* = 13, 34.2%), schematic diagrams (*n* = 11, 28.9%) or mentioned the clinical relevance of the landmarks (*n* = 13, 34.2%). For clinical relevance, most of them dealt with the maxillary sinus and the pterygomaxillary fissure (Table 2).

**Figure 3:**
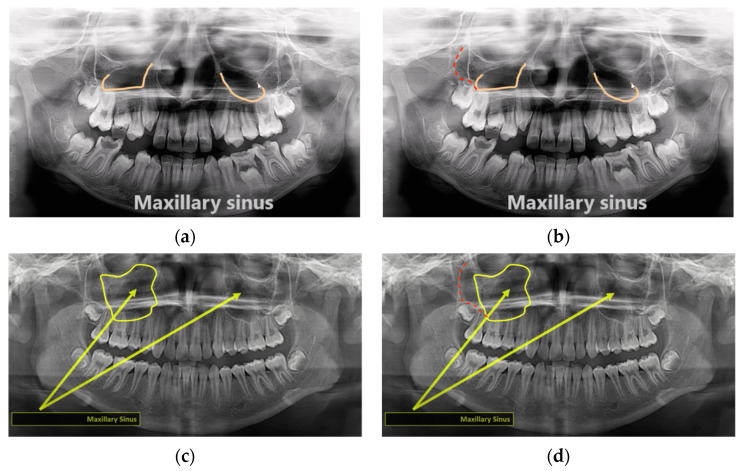
Examples of wrong delineation of the posterior wall of the maxillary sinus and their corrections. (**a**) A video wrongly treated the diagonal line due to the depression of the maxillary sinus anterior wall as the posterior sinus wall and (**b**) the correct tracing of the posterior sinus wall (red dotted line). (**c**) Another example of a video that wrongly identified the zygomatic process of the maxilla as the posterior sinus wall and (**d**) the correct tracing of the posterior sinus wall (red dotted line).

Overall, 15 videos were found to contain inaccurate descriptions of the anatomical landmarks (Table 3). Each of them contained 1–3 erroneous information. The maxillary sinus was the landmark most frequently involved in the inaccurate descriptions (Figure 3a–d).

One video might contain multiple inaccuracies.

On average, each video described 26 anatomical landmarks (Table 4), with more midfacial structures than mandibular structures. The simplest video described 4 landmarks, whereas the most comprehensive video described 48 landmarks. It should be noted that some videos formed a series by themselves (Part 1, Part 2, etc.). Since each video had its own link and viewing metrics, they were equally treated as individual videos.

**Figure 4:**
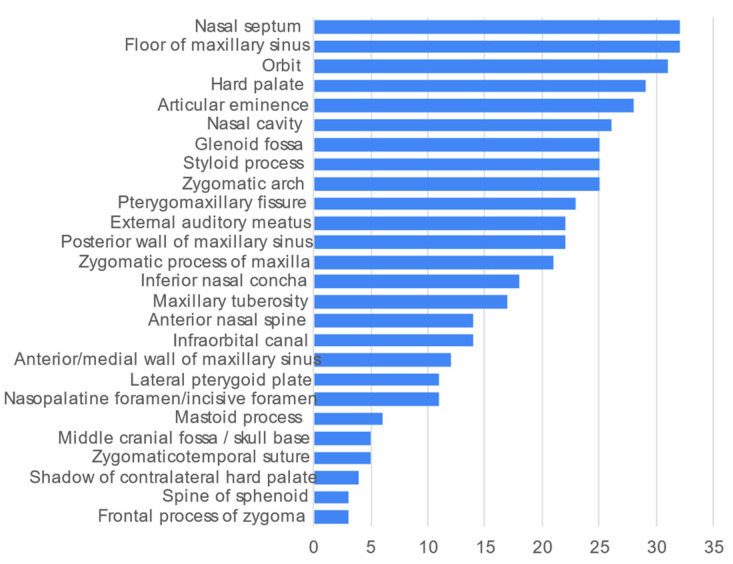
Frequency count of midfacial anatomical landmarks commonly described in the 38 videos.

The most frequently described midfacial landmarks were nasal septum and floor of maxillary sinus (*n* = 32 each, 84.2%) (Figure 4). Five structures were each described in one video only and hence not included in Figure 4, namely the lateral fossa, intermaxillary/nasopalatine suture, middle nasal concha, mastoid air cells, and sella turcica.

**Figure 5:**
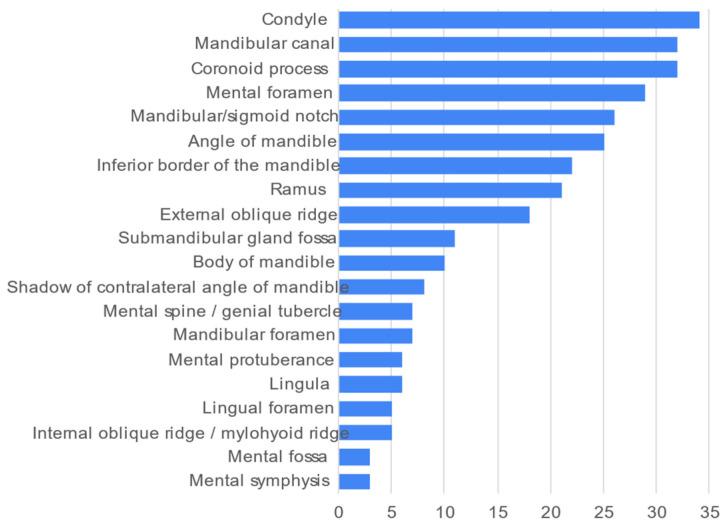
Frequency count of mandibular anatomical landmarks described in the 38 videos.

The most frequently described mandibular landmarks was the condyle of the mandible (*n* = 34, 89.5%) (Figure 5). The most seldom described landmarks were the mental symphysis and mental fossa (*n* = 3 each, 7.9%).

**Figure 6:**
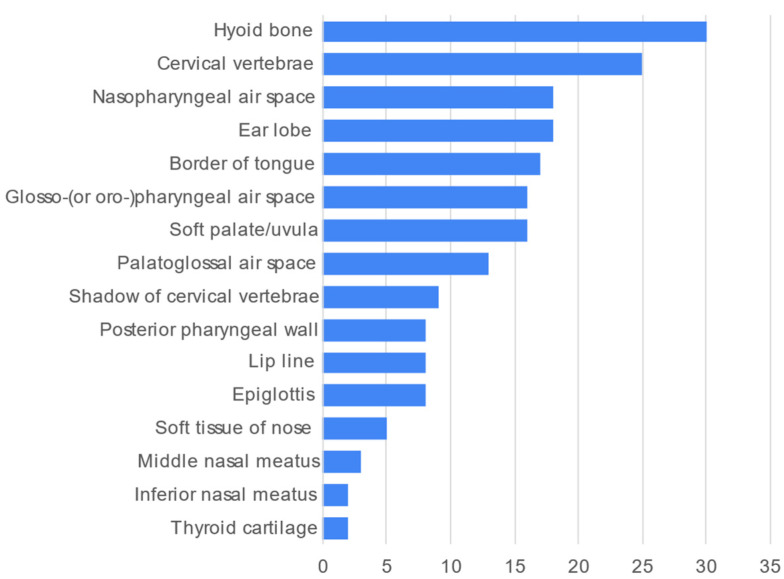
Frequency count of soft tissues/air spaces/other anatomical landmarks described in the 38 videos.

The most frequently described soft tissues/air spaces/other anatomical landmarks was the hyoid bone (Figure 6, *n* = 30, 78.9%).

Finally, it was found that view count positively correlated with like count, channel subscriber count, and video age (Table 5). Comment count, video duration, and total number of anatomical landmarks described did not correlate with view count. Meanwhile, the mean view count of videos without wrong information did not significantly differ from videos with wrong information (Mean ± SD, Without: 9569.0 ± 19,073.6, With: 4419.3 ± 10,123.8, *p* = 0.344). The same was found for the mean like count (Mean ± SD, Without: 202.0 ± 418.1, With: 81.7 ± 174.2, *p* = 0.299).

## 4. Discussion

This YouTube video survey has identified 38 videos that covered radiographic anatomy on panoramic images. Most of them showed clear panoramic images and had clear tracing or delineation of the anatomical landmarks. However, only one-third of them illustrated with the aid of skull images, rendering the audience difficult to visualize and relate to the actual corresponding structures from a three-dimensional perspective. The videos covered a wide range of anatomical structures that could be visualized by panoramic radiographs. 

Prior studies have found that nearly 80% of students in medical or healthcare programs used YouTube as a tool to learn anatomy [15,16]. If a student was not particularly fond of radiology or anatomy, the videos that covered the anatomical landmarks on a panoramic radiograph in a simplistic or mechanistic manner would be very boring and disengaging. Imagine a student who wants to learn more after class or has skipped a class and want to watch some videos as a supplement. It will be very discouraging to watch a video that numbers the anatomical landmarks randomly, names them monotonically, and finally traces them with single-coloured lines. With e-learning, the videos could be made more creative and enhanced with more animations and references to other popular videos or culture, such as the use of memes [17,18]. The video watching can be supplemented by doing interactive online exercise on the radiography anatomy on panoramic radiography (such as PanoAnatomy, https://www.dentistry.nus.edu.sg/panoanatomy/ (accessed on 1 June 2022)), so that the learnt knowledge can further be confirmed and consolidated.

It was found that only 13 of the 38 videos mentioned some points about the clinical relevance of the structures. Narration that highlights areas of clinical interest can make a video more attractive to the students [19]. It was reported that nearly all of the surveyed medical students strongly perceived the clinical relevance of anatomy knowledge [20]. In teaching dental anatomy, there was also a paradigm shift from decontextualized technical learning to more clinically applicable learning [21]. It is logical to postulate that students will pay more attention to the anatomical content if they can appreciate the relevance, or “usefulness” of the anatomical structures to their daily clinical training in terms of diagnosis, treatment, and follow-up. For instance, nasopharyngeal carcinoma may invade into the skull base or paranasal sinuses. Medial to the pterygomaxillary fissure is the pterygopalatine fossa, which provides a route for tumor to spread into the surrounding structures such as the orbit, infratemporal fossa, oral cavity, nasal cavity, and middle cranial fossa [22]. Therefore, the clinical point mentioned by some of the surveyed YouTube videos that “pterygomaxillary fissure may disappear if a maxillary lesion involves the posterior wall of the maxillary bone/sinus” is highly relevant during the evaluation of the panoramic radiographs, particularly for patients with nasopharyngeal carcinoma.

Understanding the semantic roots of the nomenclature should also help students to retain the anatomical knowledge. For instance, sella turcica is Latin and it means Turkish saddle [23]—a vivid description of the shape of the bony depression in the sphenoid bone. Similarly, the styloid process stemmed from stylus, meaning a long pen or pencil [24]. Relating to this, one issue identified from two surveyed videos was calling the zygomatic process of the maxilla simply as the “zygomatic process”. A process means a protrusion in anatomy. There are three bones that articulate with the zygomatic bone, namely the frontal bone, the maxilla, and the temporal bone. Hence, all three bones have a “zygomatic process”. In a panoramic image, the zygomatic process of the maxilla surely is the most prominent zygomatic process among the three. However, it is still necessary to name it properly to avoid confusion.

A more critical issue compared to the incomplete/inaccurate naming of the anatomical structures was the wrong identification of the structures. Four videos wrongly identified the posterior wall of the maxillary sinus, with half of them pointed at the zygomatic process of the maxilla (abovementioned) and the other half of them pointed at the diagonal line related to the depression of the anterior wall of the maxillary sinus. This depression is seldom covered by the literature, and readers can refer to [25] for its radiographic manifestation on panoramic radiography. It might be constructive for students to learn from errors, only if the errors could be identified and rectified [26]. Without a teacher sitting beside a student to point out and rectify the misinformation during the YouTube video watching, the student may digest and assimilate the misinformation unnoticed and subsequent correction could be difficult.

Though there were not many YouTube videos on radiographic anatomy on panoramic radiography, the view counts of them had a large variation. The view count only correlated to like count, channel subscriber count, and video age. In a previous study by our research team, the correlation between view count and like count was similarly reported for YouTube videos on demonstrating panoramic radiography [27]. These metrics did not necessarily reflect the content quality. In fact, the number of landmarks covered did not correlate with view count. After watching all 38 videos, the author was particularly impressed by two videos, “Anatomical Landmarks Found in a Panoramic Radiograph” (https://www.youtube.com/watch?v=Z6FTLVC6NIQ (accessed on 1 June 2022)), and “Panoramic Radiographic Anatomy” (https://www.youtube.com/watch?v=a-jrCiSrTg8 (accessed on 1 June 2022)). Their explanations were clear and easy to follow, and covered 42 and 30 landmarks, respectively. The former had 4697 views. The latter was deemed even more understandable as the landmarks were simultaneously delineated or highlighted on a skull model so that the audience could very easily relate to the skull anatomy. However, it only had 208 views. Regardless of the learning methods and resources, students and clinicians should be familiar with the radiographic anatomy and hence the normal appearance of the structures on a panoramic image, so that any deviations from the norm can be detected and recognized.

There were certain limitations of this study. Only YouTube videos narrated in English were considered. Private and unlisted videos posted on YouTube could not be accessed. The comments posted for the videos were only counted but not qualitatively evaluated. Furthermore, this study did not evaluate the frequency of using alternative names of the anatomical structures, such as inferior alveolar canal or inferior dental canal for the mandibular canal [28]. Future videos produced by educators should consider the abovementioned shortcomings to present in an interesting and error-free manner, and may even consider making interactive videos that incorporate in-video quizzes or links to related videos. 

## 5. Conclusions

Most YouTube videos showed clear panoramic images and had clear tracing or delineation of the anatomical landmarks. The videos were of good quality in general, with some frequent shortcomings being lack of visual aid with skull and schematic diagrams, and lack of discussion on clinical relevance. The maxillary sinus was the structure mostly involved in wrong information, particularly the wrong delineation of its posterior wall.

## Figures and Tables

**Table 1:** Viewing metrics of the 38 videos.

Metric	Mean ± SD	Min; Max
View count	7536.2 ± 16,174.1	35; 83,549
Like count	154.5 ± 344.9	0; 1800
Comment count	20.3 ± 56.8	0; 314
Duration (s)	892.2 ± 1138.4	60; 6333
Channel subscriber count	12,507.2 ± 37,211.5	3; 214,000
Age of video (y)	2.0 ± 1.3	0.1; 5.0

**Table 2:** Details of clinical relevance of the anatomical landmarks by the videos.

	No. of Videos	% (of 38)
**Midfacial Landmarks**		
Elongated styloid process (or calcification of stylohyoid ligament) may indicate Eagle syndome	2	5.3
Pterygomaxillary fissure may disappear if a maxillary lesion involves the posterior wall of the maxillary bone/sinus	2	5.3
Maxillary sinus will be radiopaque if pathological	1	2.6
Sinus lift is needed if an implant is placed at maxillary posterior region with a low sinus floor	1	2.6
Zygomaticotemporal suture should not be mistaken as a fracture	1	2.6
**Mandibular landmarks**		
Mandibular nerve block should be injected at mandibular foramen, and mental nerve block at mental foramen	2	5.3
Condyle may fracture upon severe trauma	1	2.6
Mental foramen should not be mistaken as a periapical lesion	1	2.6
Submandibular gland fossa should not be mistaken as a pathology such as cancer	1	2.6
**Soft tissues/air spaces/others**		
Hyoid bone should not be mistaken as the clavicle	1	2.6

**Table 3:** Details of inaccurate descriptions of the anatomical landmarks.

	No. of Videos	% (of 38)
**Midfacial landmarks**		
Named the zygomatic process of the maxilla simply as “zygomatic process”	2	5.3
Wrongly identified the zygomatic process of the maxilla as the posterior wall of maxillary sinus	2	5.3
Wrongly treated the diagonal line due to depression of the maxillary sinus anterior wall as the posterior sinus wall	2	5.3
Wrongly identified the pterygomaxillary fissure	2	5.3
Wrongly identified the zygomatic process of the maxilla	1	2.6
Wrongly identified the lateral pterygoid plate	1	2.6
Wrongly named the zygomatic process of the temporal bone as the zygomatic bone	1	2.6
**Mandibular landmarks**		
Named the mandibular notch as the coronoid notch	2	5.3
Wrongly named the condyle of the mandible as the coronoid process, and vice versa	1	2.6
Wrongly named the coronoid process as the coronoid notch	1	2.6
Called the shadow of the contralateral angle of the mandible simply as “ghost image”	1	2.6
**Soft tissues/air spaces/others**		
Grouped or named the palatoglossal air space as the glosso- (or oro-)pharynx	2	5.3
Wrongly identified the palatoglossal air space as the soft palate and uvula	1	2.6
Wrongly identified the shadow of the cervical vertebrae in the midline as the chin rest	1	2.6
Circled a line tracing the inferior border of the nasopharyngeal space and suggested that it was the whole space	1	2.6
Wrongly identified the border of the tongue	1	2.6

**Table 4:** Number of anatomical landmarks described by the 38 videos.

Type	Mean ± SD	Min; Max
Overall	25.7 ± 11.7	4; 48
Midfacial	12.3 ± 5.7	0; 22
Mandibular	8.2 ± 4.0	0; 16
Soft tissue/air space/others	5.2 ± 3.9	0; 15

**Table 5:** Pearson correlation between the viewing metrics.

	Like Count	Comment Count	Channel Subscriber Count	Duration (s)	Video Age (y)	Total no. of Landmarks
View count	0.940 (*p* < 0.001)	0.072 (*p* = 0.691)	0.788 (*p* < 0.001)	−0.121 (*p* = 0.468)	0.469 (*p* = 0.003)	−0.285 (*p* = 0.082)
Like count		0.128 (*p* = 0.478)	0.812 (*p* < 0.001)	−0.111 (*p* = 0.508)	0.245 (*p* = 0.138)	−0.221 (*p* = 0.182)
Comment count			0.018 (*p* = 0.922)	−0.133 (*p* = 0.459)	−0.191 (*p* = 0.287)	−0.199 (*p* = 0.266)
Channel subscriber count				−0.078 (*p* = 0.647)	0.169 (*p* = 0.317)	0.049 (*p* = 0.775)
Duration (s)					0.064 (*p* = 0.705)	0.241 (*p* = 0.144)
Video age						−0.153 (*p* = 0.360)

## Data Availability

All data is available in the manuscript.

## Supplementary Materials

The following supporting information can be downloaded at: https://www.mdpi.com/article/10.3390/healthcare10081382/s1, Table S1: Details of the 38 videos on the radiographic anatomy on panoramic radiography.

## References

[1] Yeung A.W.K., Tanaka R., Jacobs R., Bornstein M.M. (2020). Awareness and practice of 2D and 3D diagnostic imaging among dentists in Hong Kong. Br. Dent. J..

[2] Yeung A.W.K., Wong N.S.M. (2021). Reject Rates of Radiographic Images in Dentomaxillofacial Radiology: A Literature Review. Int. J. Environ. Res. Public Health.

[3] Hellén-Halme K., Johansson P.-M., Håkansson J., Petersson A. (2004). Image quality of digital and film radiographs in applications sent to the Dental Insurance Office in Sweden for treatment approval. Swed. Dent. J..

[4] Polo M. (2017). Bone resorption under chin implants: The orthodontist’s role in its diagnosis and management. Am. J. Orthod. Dentofacial Orthop..

[5] Yeung A.W.K., Mozos I. (2020). The innovative and sustainable use of dental panoramic radiographs for the detection of osteoporosis. Int. J. Environ. Res. Public Health.

[6] Tanaka R., Tanaka T., Yeung A.W.K., Taguchi A., Katsumata A., Bornstein M.M. (2020). Mandibular radiomorphometric indices and tooth loss as predictors for the risk of osteoporosis using panoramic radiographs. Oral Health Prev. Dent..

[7] Bruno G., De Stefani A., Balasso P., Mazzoleni S., Gracco A. (2017). Elongated styloid process: An epidemiological study on digital panoramic radiographs. J. Clin. Exp. Dent..

[8] Kaval M.E., Demirci G.K., Atesci A.A., Sarsar F., Dindaroğlu F., Güneri P., Caliskan M.K. (2022). YouTube™ as an information source for regenerative endodontic treatment procedures: Quality and content analysis. Int. J. Med. Inform..

[9] Fu M.W., Kalaichelvan A., Liebman L.S., Burns L.E. (2021). Exploring predoctoral dental student use of YouTube as a learning tool for clinical endodontic procedures. J. Dent. Educ..

[10] Aldallal S., Yates J., Ajrash M. (2019). Use of YouTube™ as a self-directed learning resource in oral surgery among undergraduate dental students: A cross-sectional descriptive study. Br. J. Oral Maxillofac. Surg..

[11] Jaffar A.A. (2012). YouTube: An emerging tool in anatomy education. Anat. Sci. Educ..

[12] Azer S.A. (2012). Can “YouTube” help students in learning surface anatomy?. Surg. Radiol. Anat..

[13] Staziaki P.V., de Oliveira Santo I.D., Skobodzinski A.A., Park L.K., Bedi H.S. (2021). How to use YouTube for radiology education. Curr. Probl. Diagn. Radiol..

[14] Kauffman L., Weisberg E.M., Eng J., Fishman E.K. (2022). YouTube and radiology: The viability, pitfalls, and untapped potential of the premier social media video platform for image-based education. Acad. Radiol..

[15] Mustafa A.G., Taha N.R., Alshboul O.A., Alsalem M., Malki M.I. (2020). Using YouTube to learn anatomy: Perspectives of Jordanian medical students. BioMed Res. Int..

[16] Barry D.S., Marzouk F., Chulak-Oglu K., Bennett D., Tierney P., O’Keeffe G.W. (2016). Anatomy education for the YouTube generation. Anat. Sci. Educ..

[17] Brown J.D. (2020). What do you meme, professor? An experiment using “memes” in pharmacy education. Pharmacy.

[18] Wells D.D. (2018). You all made dank memes: Using internet memes to promote critical thinking. J. Political Sci. Educ..

[19] Miller G.W., Lewis T.L. (2016). Anatomy education for the YouTube generation: Technical, ethical, and educational considerations. Anat. Sci. Educ..

[20] Moxham B., Plaisant O. (2007). Perception of medical students towards the clinical relevance of anatomy. Clin. Anat..

[21] Obrez A., Briggs C., Buckman J., Goldstein L., Lamb C., Knight W.G. (2011). Teaching clinically relevant dental anatomy in the dental curriculum: Description and assessment of an innovative module. J. Dent. Educ..

[22] King A.D., Bhatia K.S.S. (2010). Magnetic resonance imaging staging of nasopharyngeal carcinoma in the head and neck. World J. Radiol..

[23] Tekiner H., Acer N., Kelestimur F. (2015). Sella turcica: An anatomical, endocrinological, and historical perspective. Pituitary.

[24] Devasia B., Ranganathan K., Umadevi M., Shanmugam S., Saraswathi T. (2004). Stylocarotid syndrome mimicking dental Pain. Asian J. Oral Maxillofac. Surg..

[25] Yoshida K., Fukuda M., Gotoh K., Ariji E. (2017). Depression of the maxillary sinus anterior wall and its influence on panoramic radiography appearance. Dentomaxillofac. Radiol..

[26] Metcalfe J. (2017). Learning from errors. Annu. Rev. Psychol..

[27] Grillon M., Yeung A.W.K. (2022). Content Analysis of YouTube Videos That Demonstrate Panoramic Radiography. Healthcare.

[28] Yeung A.W.K. (2021). The Usage of the Terms Mandibular Canal, Inferior Alveolar Canal, and Inferior Dental Canal in the Academia: A Bibliometric Analysis. Int. J. Morphol..

